# 1H MR‐based detection of human plasma metabolic alterations in clear cell renal cell carcinoma

**DOI:** 10.1002/bco2.70216

**Published:** 2026-06-16

**Authors:** Raj Kumar Sharma, Meiyappan Solaiyappan, Ardit Feinaj, Stephan Brönimann, Nirmish Singla, Zaver M. Bhujwalla, Yasser Ged

**Affiliations:** ^1^ Division of Cancer Imaging Research, The Russell H. Morgan Department of Radiology and Radiological Science The Johns Hopkins University School of Medicine Baltimore Maryland USA; ^2^ Sidney Kimmel Comprehensive Cancer Center The Johns Hopkins University School of Medicine Baltimore Maryland USA; ^3^ Brady Urologic Institute The Johns Hopkins University School of Medicine Baltimore Maryland USA

**Keywords:** cancer metabolomics, MRS, plasma metabolites, RCC, small renal mass

## Abstract

**Objectives:**

This study aimed to evaluate the potential of plasma‐based proton magnetic resonance spectroscopy (1H‐MRS) for non‐invasive discrimination between clear cell renal cell carcinoma (ccRCC), benign renal masses (angiomyolipoma and oncocytoma) and healthy controls by identifying disease‐specific metabolic signatures.

**Patients and Methods:**

We performed 1H‐MRS metabolic profiling of human plasma samples from 30 individuals divided into three cohorts: ccRCC (*n* = 10, all biopsy‐confirmed, nonmetastatic T3 tumours), benign renal masses (*n* = 10, angiomyolipoma or oncocytoma) and healthy controls (*n* = 10). Multivariate and univariate statistical analyses were conducted to evaluate group separation and identify differentially abundant metabolites. Metabolite set enrichment analysis was used to identify significantly perturbed metabolic pathways associated with each state.

**Results:**

We observed altered metabolic plasma profiles in ccRCC patients compared to normal controls and benign patients. We detected significantly increased concentrations of hydroxybutyrate (normal vs. ccRCC, *p* = 0.008), glucose (normal vs. ccRCC, *p* = 0.020), (benign vs. ccRCC, *p* = 0.009), creatinine (normal vs. ccRCC, *p* = 0.015), (benign vs. ccRCC, *p* = 0.014) in ccRCC patients' plasma compared to normal controls and benign renal masses plasma. We also found that acetate and myoinositol were significantly elevated in benign (normal vs. benign, *p* = 0.0002) and ccRCC (normal vs. ccRCC, *p* = 0.0001) plasma compared to normal controls plasma. Pathway enrichment analysis revealed alterations in fatty acid biosynthesis, amino acid metabolism, nitrogen handling and glycolysis‐related pathways consistent with ccRCC‐associated metabolic reprogramming.

**Conclusion:**

This pilot study demonstrates that plasma 1H‐MRS can detect metabolic alterations associated with ccRCC and benign renal masses. These findings support the feasibility of using metabolomic profiling as a non‐invasive diagnostic tool for renal mass characterization. Larger validation studies are warranted to confirm diagnostic accuracy and explore utility in clinical decision‐making.

## 1 INTRODUCTION

Renal cell carcinoma (RCC) is a common malignancy that accounts for approximately 2%–3% of all adult cancers, with a rising global incidence partly attributable to increased use of cross‐sectional imaging and lifestyle‐related risk factors such as obesity, hypertension and smoking.^1^ RCC comprises a heterogeneous group of tumours and is broadly divided into clear cell RCC (ccRCC), constituting approximately 75% of RCC cases, with the remaining histologies (25%) collectively referred to as nonclear cell RCC (nccRCC), each associated with diverse genomic alterations and variable clinical behaviours.^2^ For patients with localized RCC, surgical resection with either partial or radical nephrectomy remains the standard curative approach, offering excellent long‐term outcomes when tumours are confined to the kidney.^3^

Despite advances in imaging modalities and surgical techniques, significant challenges remain. Notably, the reported prevalence of benign pathology among patients undergoing surgery for small renal masses varies widely, ranging from approximately 10% to 30% across contemporary cohorts, including oncocytoma and angiomyolipoma.^4^, ^5^ Although imaging can identify lipid‐rich angiomyolipomas and prevent unnecessary surgery in select cases, distinguishing benign from malignant solid renal masses remains challenging. No validated blood‐based biomarkers or regulatory‐approved diagnostic assays are currently available to guide presurgical decision‐making in this setting.

Metabolic reprogramming is a hallmark of cancer, and ccRCC is well recognized for its extensive metabolic alteration that drives tumour progression.^6^, ^7^ Cancer‐related metabolic variations and associated pathways provide the opportunity to identify tumour‐associated biomarker signatures and potentially to reveal pathway dependencies that may be therapeutically targeted. Several studies have focused on detecting molecular biomarkers from urine and plasma samples.^8^, ^9^ A recent investigation found urinary cytokines G‐CSF, GM‐CSF, IL‐6, CXCL10, CCL5 and PDGF‐BB as potential markers of tumour grade in ccRCC. Similarly, elevated plasma levels of IL‐6 and reduced concentrations of CCL2 have been observed in patients with ccRCC compared to healthy controls.^10^

Metabolomics has emerged as a powerful tool to investigate metabolic perturbations during the progression of disease. Particularly, magnetic resonance (MR) and mass‐based metabolomics approaches have been widely used to study the metabolic changes in various cancers.^11^, ^12^ Studies utilizing a metabolomics approach have shown the remarkable potential to identify ccRCC‐associated biomarkers. One such metabolomic study in ccRCC using mass spectrometry revealed that these tumours are characterized by broad shifts in central carbon metabolism, one‐carbon metabolism and antioxidant response.^13^ These metabolic changes include the accumulation of glycolytic metabolites such as glucose‐6‐phosphate, fructose‐6‐phosphate and lactate, and the depletion of fumarate and malate. The study also showed that tumour progression and metastasis were associated with increases in glutathione and cysteine/methionine metabolism pathways. Other notable metabolic alterations included glutamine, tryptophan, and fatty acid oxidation pathways. However, only a limited number of studies have explored the MR‐based metabolomics approach to identify the biomarkers for the early detection of ccRCC.

In recent years, proton magnetic resonance spectroscopy (1H‐MRS) has emerged as a non‐invasive tool to identify and quantify changes in small metabolites in various biological samples such as plasma, urine and tissue.^14^, ^15^ Although mass spectrometry provides greater analytical sensitivity, 1H‐MRS offers several practical advantages, including minimal sample preparation, high reproducibility and lower cost. These features make 1H‐MRS a complementary technique to mass spectrometry, particularly for exploratory and translational metabolomic profiling.^16^ A previous 1H‐MRS study of plasma samples obtained from a RCC patient found variations in the levels of lipoprotein, *N*‐acetylglycoproteins (NAC), lactate and choline compared to normal patient samples.^17^ A recent 1H‐MRS based metabolomic study of urine samples from normal and ccRCC patients found significant metabolic alterations in ccRCC compared to normal samples.^18^ Overall, however, plasma studies of ccRCC patients using MR metabolic profiling are limited, representing an underexplored avenue for non‐invasive biomarker discovery.

In the present study, we investigated whether plasma samples obtained from healthy controls, subjects with benign renal masses and those with ccRCC could be distinguished using 1H‐MRS metabolic profiling. Our aim was to evaluate this non‐invasive approach as a potential tool for metabolic characterization and differentiation of renal masses. We investigated the associated metabolic alterations to gain insight into dysregulated pathways involved in tumour development.

## 2 PATIENTS AND METHODS

### 2.1 Study population

This is a single‐centre study, which was conducted at the Sidney Kimmel Comprehensive Cancer Center, School of Medicine at Johns Hopkins University to evaluate the use of plasma 1H‐MRS for the detection of ccRCC as a non‐invasive liquid‐based biomarker diagnostic test. Plasma samples used in this study were collected under our Institutional Review Board‐approved biobanking protocol for Genitourinary tumours (IRB protocol # 00087094).

Plasma samples were collected with prior consent from patients undergoing nephrectomy; therefore, only patients who proceeded to surgical resection were eligible for inclusion, irrespective of final histology. Healthy control plasma samples were collected under the same protocol but were anonymized, and demographic data (e.g., age and sex) were not retained per IRB requirements for healthy volunteer biospecimen use; however, all healthy control subjects were at least 18 years of age.

Three study cohorts were included, all of whom had plasma samples collected for analysis. The ccRCC cohort consisted of participants with biopsy‐confirmed, nonmetastatic ccRCC, all with pathologic stage T3 primary tumours. The two other groups included subjects with biopsy‐confirmed benign renal masses (benign cohort) and healthy volunteers with no known renal pathology (normal cohort). For participants in the ccRCC and benign cohorts, plasma samples were collected within 4 weeks prior to surgical nephrectomy. Final histopathologic diagnoses were confirmed by a dedicated genitourinary pathologist.

### 2.2 Sample collection and plasma spectroscopy analysis

Fasting blood samples were collected from 30 human subjects enrolled in this study, including 10 participants with ccRCC (ccRCC cohort), 10 with biopsy‐confirmed benign renal masses consisting of angiomyolipoma (AML) and oncocytoma (benign cohort) and 10 healthy individuals with no known renal pathology (normal cohort).

Blood samples were obtained in heparinized syringes by standard phlebotomy technique and processed within 2 h of collection. For the isolation of plasma, blood was transferred into a 50‐mL conical tube and placed in a centrifuge at 1800 ×*g*. for 20 min with the brakes off. The plasma layer was removed and stored in 1‐mL aliquots at −80°C. Plasma samples were thawed on ice just prior to spectroscopy. Samples were prepared by mixing 350 μL of plasma with 250 μL of deuterated phosphate buffer saline to maintain the sample volume and maintain pH at 7.4 and transferred to a 5‐mm MR tube for spectroscopy.

Fully relaxed 1H MR spectra from plasma samples were acquired with a Bruker MR 750‐MHz spectrometer equipped with a triple resonance TXI probe enabled for automatic lock, tune match and shimming. Data from all the samples were acquired at room temperature. A 1D Carr–Purcell–Meiboom–Gill (CPMG, standard Bruker pulse sequence) pulse sequence was used for the data acquisition with the following parameters: number of scans 64, dummy scan 8, relaxation delay 10, receiver gain 64, and spectral width 15 ppm. All data were processed using Bruker Topspin 4.0.1. After spectral processing, all data were normalized to the external reference peak, and a variable‐size binning method was applied to calculate the area under the metabolite peak using Bruker AMIX (4.0.2) software. Metabolic assignments were performed based on previously published reports and the Biological Magnetic Resonance Bank (BMRB),^19^ Human Metabolome Data Base (HMDB).^20^

### 2.3 Statistical analyses

Baseline demographic and clinical characteristics were summarized using medians and ranges for continuous variables and frequencies and proportions for categorical variables. Histopathologic confirmation of nephrectomy specimens was performed by a dedicated genitourinary pathologist. This study was intentionally designed as an exploratory, hypothesis‐generating analysis. Therefore, no formal power calculation was performed. A sample size of 10 participants per group was chosen pragmatically to allow initial evaluation of metabolic trends and effect sizes using plasma 1H‐MRS, with the goal of informing the design of future, adequately powered validation studies.

MetaboAnalyst 6.0, a web‐based software enabled with programmes for multivariate and univariate statistical analysis, was used to perform the analysis.^21^ All bin files were exported for statistical multivariate and univariate analysis. The water region in the spectra was excluded from analysis. All data were normalized using Pareto scaling prior to multivariate statistical analysis. Supervised multivariate partial least squares‐discriminant analysis (PLS‐DA) analysis was performed on all plasma (*n* = 30) samples to examine the group classification and to extract group‐specific metabolite signatures. PLS‐DA model performance was evaluated by a model accuracy score, goodness of fit *R* ^2^ and a validation score of *Q* ^2^ Statistical analysis was performed by analysis of variance (ANOVA) followed by post hoc correction with Tukey's test for comparing multiple groups. The criterion for statistical significance was *p* < 0.05. Further, metabolite enrichment analysis was performed to identify altered groups of metabolites or biochemical pathways in normal versus benign plasma, normal versus ccRCC plasma and benign versus ccRCC plasma. This method contains a hypergeometric test that evaluates whether a particular metabolite set is represented more than expected by chance within the given compound list.^22^ In the resultant metabolite enrichment set, each metabolite set is associated with a *p*‐value, where the *p*‐value indicates the probability of observing at least a particular number of metabolites from a certain metabolite set in a given compound list. GraphPad Prism (10.4) was used for the bar plot generation.

## 3 RESULTS

Baseline characteristics of subjects in the ccRCC and benign cohorts are summarized in Table 1. Patients in the ccRCC cohort were significantly older than those in the benign group (median age: 71 vs. 58 years; *p* = 0.01). The malignant cohort had a higher proportion of male participants (70% vs. 20%; *p* = 0.05). In addition, tumour size was significantly larger in the ccRCC group compared to the benign cohort (median 9 cm vs. 3 cm, *p* = 0.0007). Among the malignant tumours in the ccRCC, 70% were staged as pT3a, 20% as pT3b and 10% as pT3c. Tumour grade distribution within the malignant cohort was as follows: 10% Grade 2, 80% Grade 3 and 10% Grade 4; with no cases classified as Grade 1. The benign lesions in the benign cohort were evenly divided between angiomyolipoma (50%) and oncocytoma (50%).

**TABLE 1:** The demographic details of human participants and tumour features in the ccRCC and benign cohorts.

	ccRCC cohort (*n* = 10)	Benign cohort (*n* = 10)	*p*‐value
**Age (years)**	71 (52–81)	58 (33–65)	0.01
**Sex**			
Female	3 (30%)	8 (80%)	0.05
Male	7 (70%)	2 (20%)	
**Histology**			
Clear cell RCC	10 (100%)	0	
AML	0	5 (50%)	
Oncocytoma	0	5 (50%)	
**Primary tumour grade**			
1	0	NA	
2	1 (10%)	NA	
3	8 (80%)	NA	
4	1 (10%)	NA	
**Primary tumour size (cm)**	9.0 (5.0–12.5)	3 (1.6–10)	0.0007
**Primary tumour stage**			
pT3a	7(70%)	NA	
pT3b	2 (20%)	NA	
pT3c	1 (10%)	NA	

### 3.1 1H MR spectroscopy of plasma samples

**FIGURE 1:**
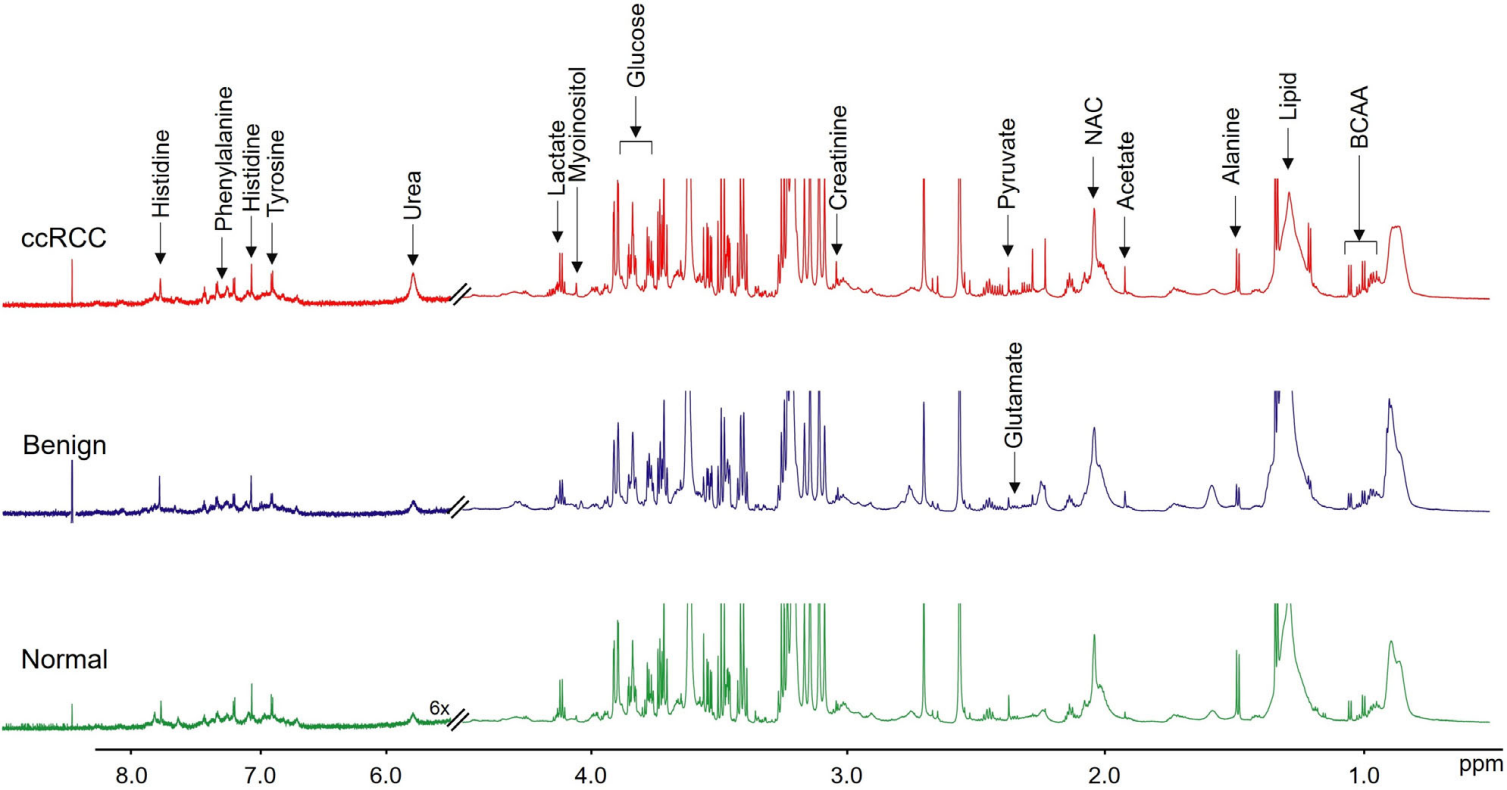
Representative high‐resolution 1H MR (CPMG) spectra obtained from normal (green), benign (blue), and ccRCC (red) human plasma samples along with the corresponding metabolite assignments. All MR spectra were plotted on the same vertical scale and acquired with similar parameters: a 90° flip angle, spectral width 15 495 Hz width, block size 64 k, eight dummy scan, 64 scans, relaxation delay 10 s, receiver gain 64 and an acquisition time of 2.11 s.

1H MR spectra obtained from normal, benign and ccRCC patients' plasma were analysed. Representative 1H MR spectra with corresponding metabolic signatures of normal, benign and ccRCC human plasma samples are shown in Figure 1. Also displayed are peak assignments of the important metabolites such as leucine, isoleucine, valine, hydroxybutyrate, lactate, alanine, acetate, glucose, pyruvate, citrate, creatinine, lactate, myoinositol, glucose, urea and aromatic amino acids. Spectral analysis was aimed at identifying specific metabolic differences in plasma from ccRCC patients compared with benign renal masses and healthy controls. Inspection of the comparison of plasma spectra identified differences in specific metabolites. However, to confirm these changes and for further analysis, we performed multivariate statistical analysis to gain further insights from the data.

### 3.2 Metabolic profile‐based discrimination between normal, benign and ccRCC plasma samples

**FIGURE 2:**
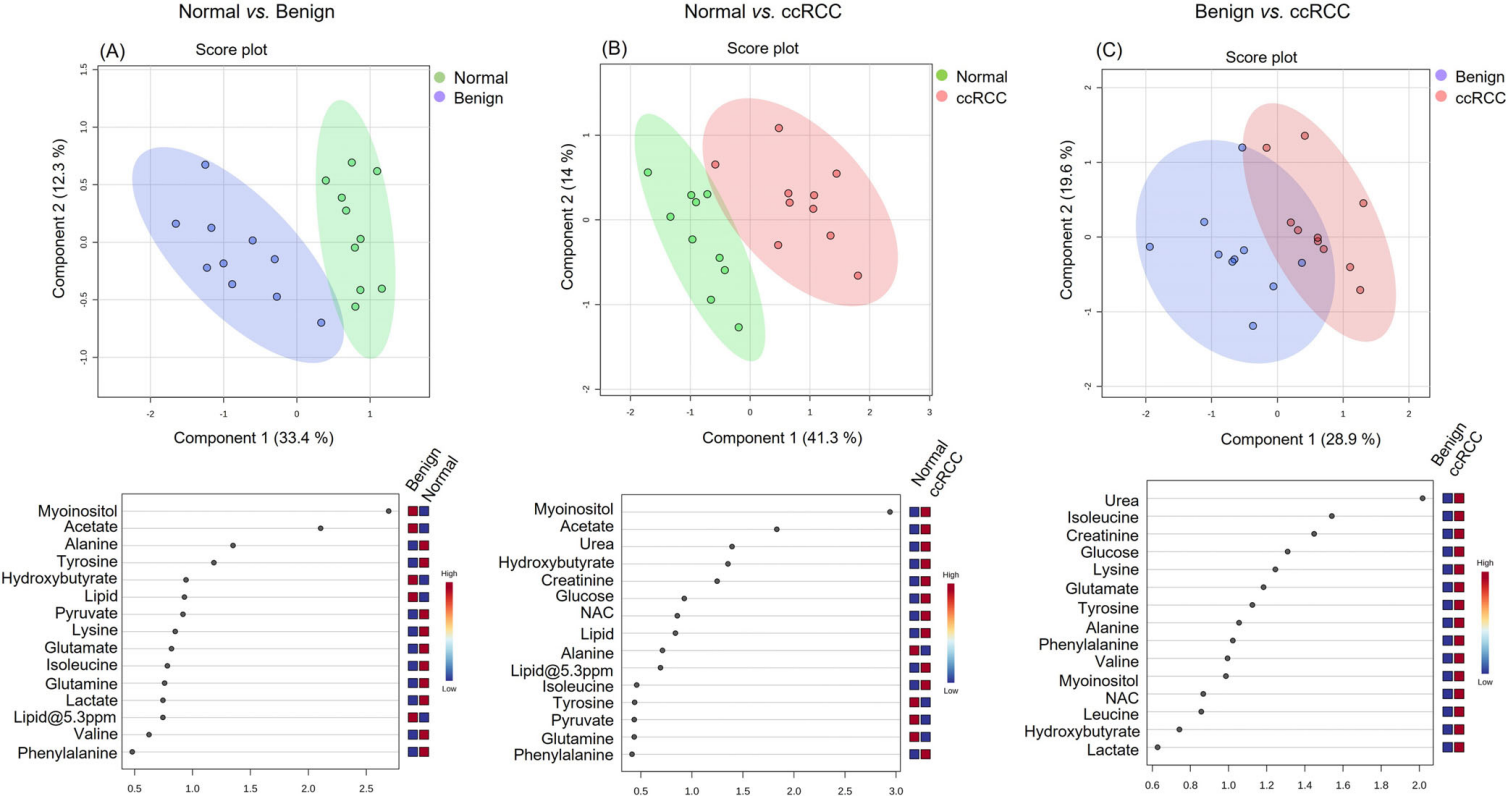
Multivariate partial least squares‐discriminant analysis (PLS‐DA) of 1H MR spectra of plasma showing clustering of groups. Score plots are shown on top with the corresponding variable importance on the projection (VIP) plot on the bottom. (A) Normal vs. benign, (B) Normal vs. ccRCC, (C) benign vs. ccRCC.

A supervised pairwise PLS‐DA analysis was performed to derive the important information based on the discriminant metabolic profile of the data. This approach enabled us to see the overall group pattern and clustering trends among the plasma samples of normal, benign and ccRCC patients, highlighting the key metabolites responsible for group separation. Score plots obtained from pairwise PLS‐DA analysis of each group are shown along with the corresponding variable importance plot (VIP) showing the involvement of key metabolites for the group discrimination in Figure 2A–C. These data identify differences in the metabolic profile, highlighting the discriminatory importance of metabolites.

**FIGURE 3:**
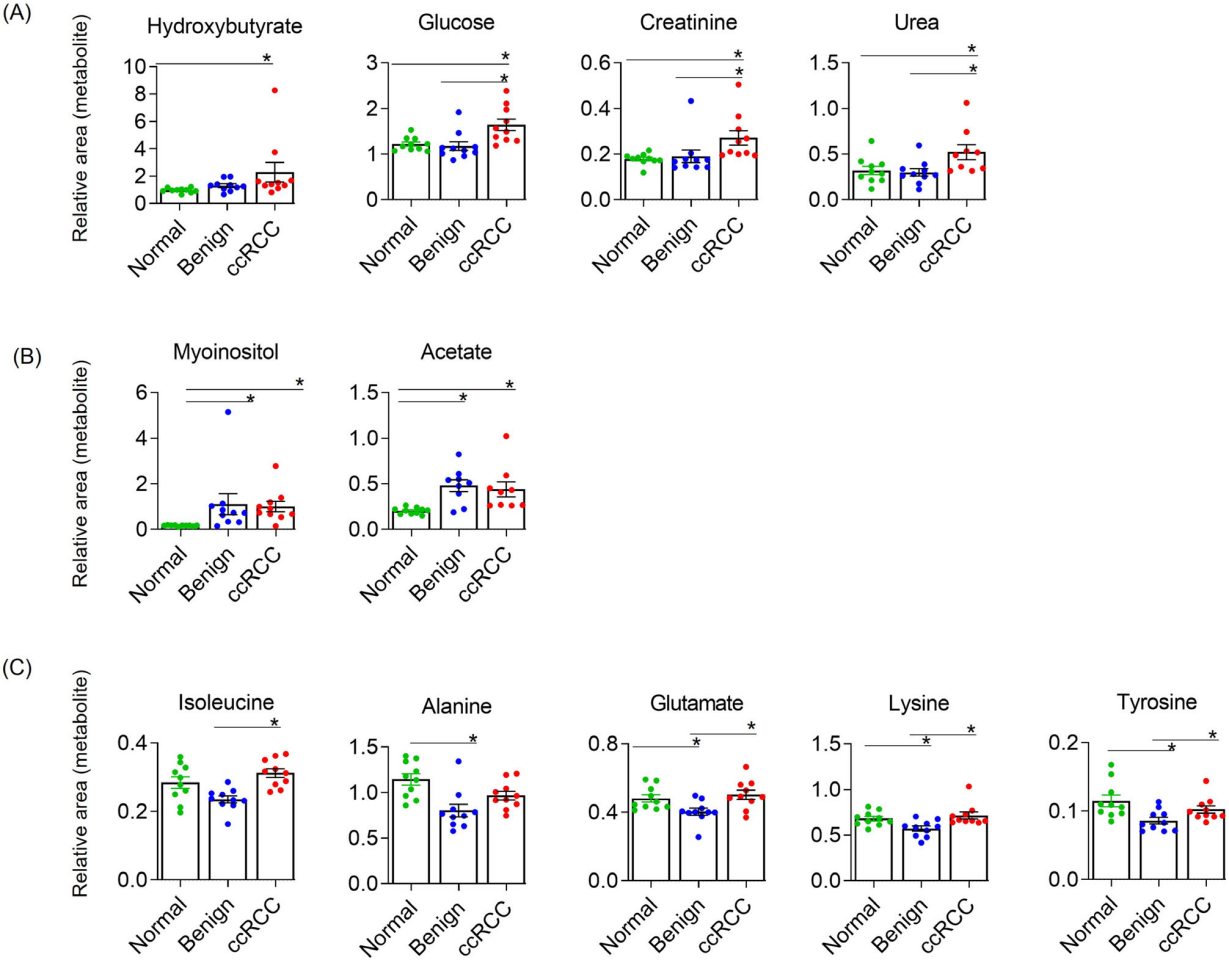
Quantitative estimation of metabolites in human plasma of normal (*n* = 10), benign (*n* = 10) and ccRCC (*n* = 10) patients. (A) Metabolites that were significantly higher in ccRCC plasma compared to normal and benign plasma. (B) Metabolites that were significantly higher in benign and ccRCC plasma compared to normal plasma. (C) Metabolites that were significantly higher in ccRCC plasma compared to benign plasma. Statistical significance was assessed by *p*‐values <0.05, calculated by analysis of variance (ANOVA). Bar plots represent mean ± SEM. Areas under the peaks were normalized to references. * represents *p* ≤ 0.05.

We found a strong separation between normal versus benign plasma samples with a good accuracy and predictability score of the PLS‐DA model (*R* ^2^ = 0.78 and *Q* ^2^ = 0.67). Subsequently, statistical analysis was performed to select the significantly altered metabolites. Elevated levels of myoinositol and acetate were detected in benign plasma compared to normal plasma. We also detected decreased levels of alanine, glutamate, lysine and tyrosine in benign plasma compared to normal plasma. Figure 3A–C.

PLS‐DA analysis demonstrated good separation between normal vs. ccRCC plasma samples with good accuracy and predictability of the model (*R* ^2^ = 0.70 and *Q* ^2^ = 0.62). Based on the statistical analysis, we detected elevated levels of hydroxybutyrate, creatinine, glucose, urea, myoinositol and acetate in ccRCC plasma compared to normal plasma Figure 3A,B.

Benign and ccRCC plasma samples exhibited clustering with a partial overlap with a model accuracy of (*R* ^2^ = 0.59 and *Q* ^2^ = 0.40). Further statistical analysis revealed significantly elevated hydroxybutyrate, creatinine, glucose, urea, myoinositol, acetate, isoleucine, glutamate, lysine and tyrosine in ccRCC plasma compared to benign plasma as shown in Figure 3A–C. No significant decrease of metabolites was found in ccRCC plasma compared to benign plasma.

### 3.3 Metabolite set enrichment analysis

Metabolite set enrichment analysis revealed significantly altered metabolic pathways in benign and ccRCC cases. Significantly altered metabolites were selected to identify metabolically affected pathways. Metabolic reprogramming was evident in the transition from normal to benign and ccRCC stages. Figure S1A–C highlight the top key metabolic pathways involved. In normal versus benign patient plasma, we found major alterations in pathways associated with amino acid metabolism that involve the glucose‐alanine cycle and alanine metabolism (Table S1). Analysis of normal versus ccRCC plasma showed fatty acid biosynthesis, galactose metabolism and lactose degradation were significantly altered (Table S2). A comparison of benign versus ccRCC plasma identified alterations in the glucose‐alanine cycle, phenylalanine and tyrosine metabolism, urea cycle, lysine degradation, arginine and proline metabolism, Warburg effect, valine, leucine, isoleucine degradation and biotin metabolism (Table S3).

## 4 DISCUSSION

In this study, we investigated the utility of plasma 1H‐MRS for non‐invasive discrimination between patients presenting with a renal mass suspicious for ccRCC and those with benign renal masses (oncocytoma and angiomyolipoma) with a normal healthy control cohort. Our findings demonstrate that MR‐based metabolomic profiling of plasma can distinguish ccRCC from both benign and normal conditions, with distinct metabolic signatures associated with each state.

A subset of patients presenting with renal masses ultimately harbour benign pathology, and current imaging modalities are often insufficient to reliably differentiate these cases.^4^ As a result, many patients undergo unnecessary surgical intervention. There are currently no FDA‐approved biomarkers for non‐invasive discrimination of benign from malignant renal masses. Recently, circulating kidney injury molecule‐1 (KIM‐1) gained momentum as a promising circulating biomarker, which can be detected in plasma and urine.^23^, ^24^ In a study of two independent cohorts of adults with renal masses, plasma KIM‐1 distinguished RCC versus benign masses with AUROC 0.81 in Cohort 1, and AUROC 0.74 in Cohort 2.^25^ Furthermore, recent functional imaging studies showed promising results in the discrimination between malignant and benign renal masses. The ZIRCON trial, which evaluated ^89^Zr‐girentuximab PET/CT, a carbonic anhydrase‐9 radioligand, demonstrated high sensitivity (85.5%) and specificity (89.5%) in small renal tumours as a potential non‐invasive tool for differentiating malignant from benign renal masses.^26^ Another promising modality is ^99^mTc‐sestamibi SPECT/CT, which has shown high specificity for oncocytomas and hybrid oncocytic/chromophobe tumours.^27^ Our study builds on these concepts by evaluating plasma 1H‐MRS as a non‐invasive diagnostic modality, leveraging the metabolic reprogramming characteristic of ccRCC.

Our multivariate analyses revealed distinct metabolic separation between normal and disease groups. The strongest discrimination was observed between normal and benign samples (PLS‐DA *R* ^2^ = 0.78, *Q* ^2^ = 0.67) and between normal and ccRCC samples (*R* ^2^ = 0.70, *Q* ^2^ = 0.62). These models identified several discriminatory metabolites, including elevated levels of myoinositol and acetate in benign plasma and increased levels of hydroxybutyrate, creatinine, glucose, urea and branch chain amino acids (BCAAs) in ccRCC plasma. The comparison between benign and ccRCC cohorts showed moderate separation (*R* ^2^ = 0.59, *Q* ^2^ = 0.40), with ccRCC plasma enriched in a broad array of metabolites associated with altered energy metabolism, nitrogen handling and amino acid turnover.

These results are consistent with prior studies describing the extensive metabolic reprogramming that accompanies ccRCC pathogenesis. It is known that pseudohypoxia driven by inactivation of the von Hippel–Lindau (VHL) gene and sustained hypoxia‐inducible factor (HIF) signalling leads to a shift away from oxidative phosphorylation and toward glycolysis and reductive glutamine metabolism.^28^ Our observation of elevated glucose, hydroxybutyrate and amino acids in ccRCC plasma is consistent with the metabolic shift toward glycolysis and altered amino acid metabolism. Moreover, the elevated levels of glutamate, tyrosine, lysine and BCAAs in ccRCC patients reflect upregulation of amino acid catabolism and transamination pathways as key features of metabolic rewiring in aggressive tumours.

Metabolite set enrichment analysis further supported these findings, revealing pathway‐level disruptions consistent with known biological mechanisms. In benign versus normal comparisons, altered glucose‐alanine cycle and alanine metabolism pathways were observed. In ccRCC plasma, we identified enrichment in fatty acid biosynthesis, galactose metabolism and lactose degradation, pathways previously implicated in ccRCC metabolic shifts. The benign versus ccRCC comparison revealed extensive alterations in amino acid and nitrogen handling pathways, including phenylalanine and tyrosine metabolism, lysine degradation, urea cycle and the Warburg effect. These findings align with recent tumour tissue studies and flux tracing experiments showing that ccRCC tumours preferentially route glutamine‐derived carbon toward lipid biosynthesis and away from full TCA cycle oxidation.^13^, ^29^ Of note, the malignant cohort included larger tumours than the benign cohort, raising the possibility that some metabolic alterations may reflect tumour burden or systemic physiological effects rather than purely tumour‐intrinsic metabolism. For example, elevated creatinine and urea levels observed in ccRCC patients likely reflect differences in renal function, consistent with impaired nitrogen handling and kidney physiology, whereas other metabolites such as amino acids and hydroxybutyrate map to metabolic pathways previously implicated in ccRCC biology. However, given the small sample size, our study was not powered to assess metabolite–tumour size correlations.

Our study has several limitations. The sample size was small (*n* = 10 per cohort), which limits statistical power, particularly in supervised models like PLS‐DA. Additionally, although 1H‐MRS offers advantages such as minimal sample preparation and reproducibility, it has lower sensitivity than mass spectrometry, potentially limiting detection of low‐abundance metabolites. Differences in age between the malignant and benign cohorts may have introduced confounding, as certain metabolites can vary with age; however, the exploratory nature of the study and limited sample sizes precluded age‐adjusted analyses. Furthermore, demographic data for healthy controls were not available due to anonymization requirements, which limited demographic comparison across cohorts, although all healthy control subjects were at least 18 years of age. Finally, our study focused exclusively on pathologic T3 ccRCC tumours; whether these metabolic signatures apply to earlier stages or other RCC subtypes remains to be determined.

In conclusion, this exploratory analysis demonstrates that plasma 1H‐MRS metabolomic profiling may capture metabolic alterations associated with ccRCC. Future studies with larger, more diverse cohorts and external validation will be required to determine whether these metabolic signatures may have clinical utility, either alone or in combination with other diagnostic approaches.

## AUTHOR CONTRIBUTIONS

**Raj Kumar Sharma:** Study design; data collection; data analysis; manuscript writing and approval. **Meiyappan Solaiyappan:** Study design; data analysis; manuscript writing and approval. **Ardit Feinaj:** Data collection; manuscript writing and approval. **Stephan Brönimann:** Data collection; manuscript writing and approval. **Nirmish Singla:** Study design; data collection; data analysis; funding; manuscript writing and approval. **Zaver M Bhujwalla:** Study design; data collection; data analysis; funding; manuscript writing and approval. **Yasser Ged:** Study design; data collection; data analysis; funding; manuscript writing and approval.

## CONFLICTS OF INTEREST STATEMENT

The authors declare no conflicts of interest.

## Supporting information


**Table S1:** Comparison between the Normal Cohort vs Benign Cohort Plasma Metabolites.

**Table S2:** Comparison between the Normal Cohort vs Clear Cell RCC Cohort Plasma Metabolites.

**Table S3:** Comparison between the Benign Cohort vs Clear Cell RCC Cohorts Plasma Metabolites.

**Figure S1.** Overview of the Top Enriched Metabolite Sets. (A) normal vs. benign plasma, (B) normal vs. ccRCC plasma, and (C) benign vs. ccRCC plasma. The bubble plot colour represents statistical significance and the bubble size shows the enrichment ratio. Metabolite enrichment analysis was performed using significantly altered (*p* < 0.05) metabolites.
