# Supplementary material for: 1H MR‐based detection of human plasma metabolic alterations in clear cell renal cell carcinoma
Source: BJUI Compass. 2026 Jun 16;7(6):e70216. doi: 10.1002/bco2.70216 (PMC13270396; doi:10.1002/bco2.70216)
Supplement: Supplementary file 1 — Table S1: Comparison between the Normal Cohort vs Benign Cohort Plasma Metabolites. [file BCO2-7-e70216-s001.docx]

**Supplementary Table1: Comparison between the Normal Cohort vs Benign Cohort Plasma Metabolites**

| Metabolite Set Pathway | Total Metabolites^1^ | Expected Hits^2^ | Observed Hits^3^ | Raw p | Adjusted p-value (Holm)^4^ | FDR^4^ |
| --- | --- | --- | --- | --- | --- | --- |
| Glucose-Alanine Cycle | 13 | 0.0778 | 2 | 0.00227 | 0.222 | 0.177 |
| Alanine Metabolism | 17 | 0.102 | 2 | 0.00391 | 0.379 | 0.177 |
| Glutathione Metabolism | 20 | 0.12 | 2 | 0.00542 | 0.52 | 0.177 |
| Phenylalanine and Tyrosine Metabolism | 27 | 0.162 | 2 | 0.00982 | 0.933 | 0.197 |
| Urea Cycle | 28 | 0.168 | 2 | 0.0105 | 0.991 | 0.197 |
| Lysine Degradation | 30 | 0.18 | 2 | 0.0121 | 1.0 | 0.197 |
| Amino Sugar Metabolism | 33 | 0.198 | 2 | 0.0145 | 1.0 | 0.2 |
| Aspartate Metabolism | 35 | 0.21 | 2 | 0.0163 | 1.0 | 0.2 |
| Glutamate Metabolism | 48 | 0.287 | 2 | 0.0298 | 1.0 | 0.325 |
| Glycine and Serine Metabolism | 59 | 0.353 | 2 | 0.0439 | 1.0 | 0.384 |
| Tryptophan Metabolism | 59 | 0.353 | 2 | 0.0439 | 1.0 | 0.384 |
| Biotin Metabolism | 8 | 0.0479 | 1 | 0.0471 | 1.0 | 0.384 |
| Malate-Aspartate Shuttle | 10 | 0.0599 | 1 | 0.0585 | 1.0 | 0.421 |
| Tyrosine Metabolism | 70 | 0.419 | 2 | 0.0601 | 1.0 | 0.421 |
| Thyroid hormone synthesis | 13 | 0.0778 | 1 | 0.0755 | 1.0 | 0.494 |
| Phosphatidylinositol Phosphate Metabolism | 17 | 0.102 | 1 | 0.0978 | 1.0 | 0.599 |
| Ethanol Degradation | 19 | 0.114 | 1 | 0.109 | 1.0 | 0.622 |
| Catecholamine Biosynthesis | 20 | 0.12 | 1 | 0.114 | 1.0 | 0.622 |
| Carnitine Synthesis | 22 | 0.132 | 1 | 0.125 | 1.0 | 0.645 |
| Inositol Phosphate Metabolism | 24 | 0.144 | 1 | 0.136 | 1.0 | 0.665 |
| Cysteine Metabolism | 26 | 0.156 | 1 | 0.146 | 1.0 | 0.674 |
| Selenoamino Acid Metabolism | 27 | 0.162 | 1 | 0.152 | 1.0 | 0.674 |
| Folate Metabolism | 29 | 0.174 | 1 | 0.162 | 1.0 | 0.674 |
| Inositol Metabolism | 30 | 0.18 | 1 | 0.167 | 1.0 | 0.674 |
| Ammonia Recycling | 31 | 0.186 | 1 | 0.172 | 1.0 | 0.674 |
| Beta-Alanine Metabolism | 34 | 0.204 | 1 | 0.188 | 1.0 | 0.674 |
| Nicotinate and Nicotinamide Metabolism | 35 | 0.21 | 1 | 0.193 | 1.0 | 0.674 |
| Fatty Acid Biosynthesis | 35 | 0.21 | 1 | 0.193 | 1.0 | 0.674 |
| Galactose Metabolism | 38 | 0.228 | 1 | 0.208 | 1.0 | 0.701 |
| Propanoate Metabolism | 42 | 0.251 | 1 | 0.227 | 1.0 | 0.718 |
| Histidine Metabolism | 42 | 0.251 | 1 | 0.227 | 1.0 | 0.718 |
| Pyruvate Metabolism | 47 | 0.281 | 1 | 0.251 | 1.0 | 0.769 |
| Arginine and Proline Metabolism | 52 | 0.311 | 1 | 0.274 | 1.0 | 0.814 |
| Warburg Effect | 57 | 0.341 | 1 | 0.297 | 1.0 | 0.856 |
| Valine, Leucine and Isoleucine Degradation | 59 | 0.353 | 1 | 0.306 | 1.0 | 0.856 |
| Arachidonic Acid Metabolism | 67 | 0.401 | 1 | 0.341 | 1.0 | 0.927 |
| Purine Metabolism | 73 | 0.437 | 1 | 0.366 | 1.0 | 0.968 |
| 1. number of metabolites included in the pathway database.  2. number of metabolites expected in the pathway by chance.  3. number of metabolites from the study dataset mapped to the pathway  4. Holm-adjusted p-values and false discovery rate (FDR) account for multiple comparisons. | | | | | | |
