# Supplementary material for: 1H MR‐based detection of human plasma metabolic alterations in clear cell renal cell carcinoma
Source: BJUI Compass. 2026 Jun 16;7(6):e70216. doi: 10.1002/bco2.70216 (PMC13270396; doi:10.1002/bco2.70216)
Supplement: Supplementary file 2 — Table S2: Comparison between the Normal Cohort vs Clear Cell RCC Cohort Plasma Metabolites. [file BCO2-7-e70216-s002.docx]

**Supplementary Table 2: Comparison between the Normal Cohort vs Clear Cell RCC Cohort Plasma Metabolites**

| Metabolite Set Pathway | Total Metabolites^1^ | Expected Hits^2^ | Observed Hits^3^ | Raw p | Adjusted p-value (Holm)^4^ | FDR^4^ |
| --- | --- | --- | --- | --- | --- | --- |
| Fatty Acid Biosynthesis | 35 | 0.175 | 2 | 0.0111 | 1 | 0.639 |
| Galactose Metabolism | 38 | 0.19 | 2 | 0.013 | 1 | 0.639 |
| Lactose Degradation | 9 | 0.0449 | 1 | 0.0442 | 1 | 0.862 |
| D-Arginine and D-Ornithine Metabolism | 11 | 0.0549 | 1 | 0.0538 | 1 | 0.862 |
| Trehalose Degradation | 11 | 0.0549 | 1 | 0.0538 | 1 | 0.862 |
| Ketone Body Metabolism | 13 | 0.0649 | 1 | 0.0633 | 1 | 0.862 |
| Glucose-Alanine Cycle | 13 | 0.0649 | 1 | 0.0633 | 1 | 0.862 |
| Phosphatidylinositol Phosphate Metabolism | 17 | 0.0848 | 1 | 0.0822 | 1 | 0.862 |
| Lactose Synthesis | 19 | 0.0948 | 1 | 0.0915 | 1 | 0.862 |
| Ethanol Degradation | 19 | 0.0948 | 1 | 0.0915 | 1 | 0.862 |
| Transfer of Acetyl Groups into Mitochondria | 22 | 0.11 | 1 | 0.105 | 1 | 0.862 |
| Glycolysis | 23 | 0.115 | 1 | 0.11 | 1 | 0.862 |
| Inositol Phosphate Metabolism | 24 | 0.12 | 1 | 0.114 | 1 | 0.862 |
| Urea Cycle | 28 | 0.14 | 1 | 0.132 | 1 | 0.888 |
| Inositol Metabolism | 30 | 0.15 | 1 | 0.141 | 1 | 0.888 |
| Amino Sugar Metabolism | 33 | 0.165 | 1 | 0.154 | 1 | 0.888 |
| Gluconeogenesis | 33 | 0.165 | 1 | 0.154 | 1 | 0.888 |
| Aspartate Metabolism | 35 | 0.175 | 1 | 0.163 | 1 | 0.888 |
| Sphingolipid Metabolism | 40 | 0.2 | 1 | 0.185 | 1 | 0.952 |
| Pyruvate Metabolism | 47 | 0.235 | 1 | 0.214 | 1 | 1 |
| Arginine and Proline Metabolism | 52 | 0.259 | 1 | 0.234 | 1 | 1 |
| Warburg Effect | 57 | 0.284 | 1 | 0.254 | 1 | 1 |
| 1. number of metabolites included in the pathway database.  2. number of metabolites expected in the pathway by chance.  3. number of metabolites from the study dataset mapped to the pathway  4. Holm-adjusted p-values and false discovery rate (FDR) account for multiple comparisons. | | | | | | |
