# Supplementary material for: 1H MR‐based detection of human plasma metabolic alterations in clear cell renal cell carcinoma
Source: BJUI Compass. 2026 Jun 16;7(6):e70216. doi: 10.1002/bco2.70216 (PMC13270396; doi:10.1002/bco2.70216)
Supplement: Supplementary file 3 — Table S3: Comparison between the Benign Cohort vs Clear Cell RCC Cohorts Plasma Metabolites. [file BCO2-7-e70216-s003.docx]

**Supplementary Table 3: Comparison between the Benign Cohort vs Clear Cell RCC Cohorts Plasma Metabolites**

| Metabolite Set Pathway | Total Metabolites^1^ | Expected Hits^2^ | Observed Hits^3^ | Raw p | Adjusted p-value (Holm)^4^ | FDR^4^ |
| --- | --- | --- | --- | --- | --- | --- |
| Glucose-Alanine Cycle | 13 | 0.0778 | 2 | 0.00227 | 0.222 | 0.222 |
| Phenylalanine and Tyrosine Metabolism | 27 | 0.162 | 2 | 0.00982 | 0.952 | 0.296 |
| Urea Cycle | 28 | 0.168 | 2 | 0.0105 | 1 | 0.296 |
| Lysine Degradation | 30 | 0.18 | 2 | 0.0121 | 1 | 0.296 |
| Arginine and Proline Metabolism | 52 | 0.311 | 2 | 0.0347 | 1 | 0.484 |
| Warburg Effect | 57 | 0.341 | 2 | 0.0411 | 1 | 0.484 |
| Valine, Leucine and Isoleucine Degradation | 59 | 0.353 | 2 | 0.0439 | 1 | 0.484 |
| Biotin Metabolism | 8 | 0.0479 | 1 | 0.0471 | 1 | 0.484 |
| Lactose Degradation | 9 | 0.0539 | 1 | 0.0528 | 1 | 0.484 |
| Malate-Aspartate Shuttle | 10 | 0.0599 | 1 | 0.0585 | 1 | 0.484 |
| Tyrosine Metabolism | 70 | 0.419 | 2 | 0.0601 | 1 | 0.484 |
| D-Arginine and D-Ornithine Metabolism | 11 | 0.0659 | 1 | 0.0642 | 1 | 0.484 |
| Trehalose Degradation | 11 | 0.0659 | 1 | 0.0642 | 1 | 0.484 |
| Thyroid hormone synthesis | 13 | 0.0778 | 1 | 0.0755 | 1 | 0.529 |
| Alanine Metabolism | 17 | 0.102 | 1 | 0.0978 | 1 | 0.608 |
| Lactose Synthesis | 19 | 0.114 | 1 | 0.109 | 1 | 0.608 |
| Catecholamine Biosynthesis | 20 | 0.12 | 1 | 0.114 | 1 | 0.608 |
| Glutathione Metabolism | 20 | 0.12 | 1 | 0.114 | 1 | 0.608 |
| Carnitine Synthesis | 22 | 0.132 | 1 | 0.125 | 1 | 0.608 |
| Transfer of Acetyl Groups into Mitochondria | 22 | 0.132 | 1 | 0.125 | 1 | 0.608 |
| Glycolysis | 23 | 0.138 | 1 | 0.13 | 1 | 0.608 |
| Cysteine Metabolism | 26 | 0.156 | 1 | 0.146 | 1 | 0.651 |
| Folate Metabolism | 29 | 0.174 | 1 | 0.162 | 1 | 0.651 |
| Ammonia Recycling | 31 | 0.186 | 1 | 0.172 | 1 | 0.651 |
| Amino Sugar Metabolism | 33 | 0.198 | 1 | 0.182 | 1 | 0.651 |
| Gluconeogenesis | 33 | 0.198 | 1 | 0.182 | 1 | 0.651 |
| Beta-Alanine Metabolism | 34 | 0.204 | 1 | 0.188 | 1 | 0.651 |
| Nicotinate and Nicotinamide Metabolism | 35 | 0.21 | 1 | 0.193 | 1 | 0.651 |
| Aspartate Metabolism | 35 | 0.21 | 1 | 0.193 | 1 | 0.651 |
| Galactose Metabolism | 38 | 0.228 | 1 | 0.208 | 1 | 0.674 |
| Sphingolipid Metabolism | 40 | 0.24 | 1 | 0.217 | 1 | 0.674 |
| Propanoate Metabolism | 42 | 0.251 | 1 | 0.227 | 1 | 0.674 |
| Histidine Metabolism | 42 | 0.251 | 1 | 0.227 | 1 | 0.674 |
| Glutamate Metabolism | 48 | 0.287 | 1 | 0.256 | 1 | 0.737 |
| Glycine and Serine Metabolism | 59 | 0.353 | 1 | 0.306 | 1 | 0.833 |
| Tryptophan Metabolism | 59 | 0.353 | 1 | 0.306 | 1 | 0.833 |
| Arachidonic Acid Metabolism | 67 | 0.401 | 1 | 0.341 | 1 | 0.902 |
| Purine Metabolism | 73 | 0.437 | 1 | 0.366 | 1 | 0.943 |
| 1. number of metabolites included in the pathway database.  2. number of metabolites expected in the pathway by chance.  3. number of metabolites from the study dataset mapped to the pathway  4. Holm-adjusted p-values and false discovery rate (FDR) account for multiple comparisons. | | | | | | |
