# Supplementary figures and images for: 1H MR‐based detection of human plasma metabolic alterations in clear cell renal cell carcinoma
Source: BJUI Compass. 2026 Jun 16;7(6):e70216. doi: 10.1002/bco2.70216 (PMC13270396; doi:10.1002/bco2.70216)

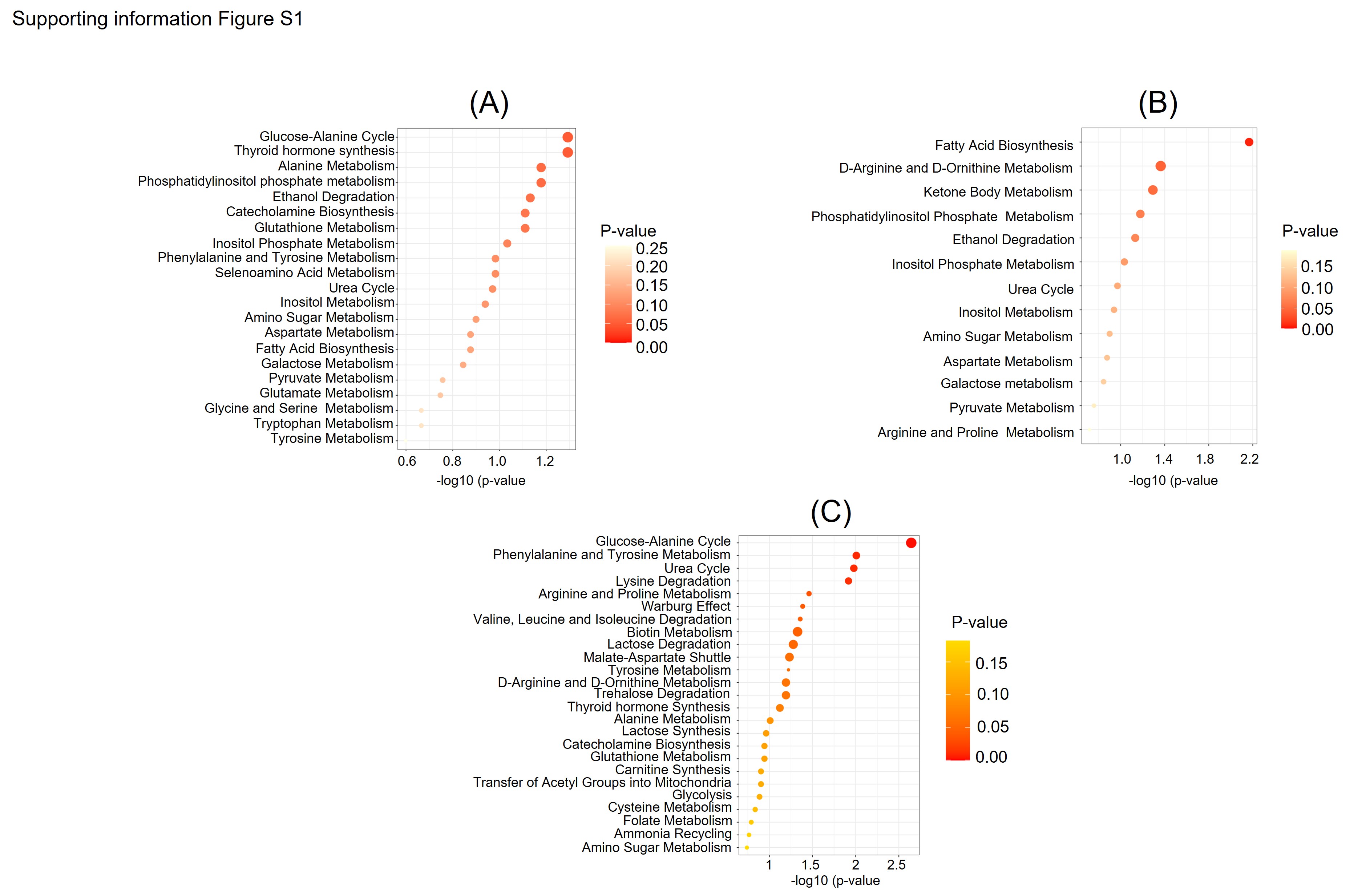

Supplement: Supplementary file 4 — Figure S1. Overview of the Top Enriched Metabolite Sets. (A) normal vs. benign plasma, (B) normal vs. ccRCC plasma, and (C) benign vs. ccRCC plasma. The bubble plot colour represents statistical significance and the bubble size shows the enrichment ratio. Metabolite enrichment analysis was performed using significantly altered (p < 0.05) metabolites. [file BCO2-7-e70216-s004.jpg]
